# In vitro evaluation of antimicrobial effect of miswak against common oral pathogens

**DOI:** 10.12669/pjms.302.4284

**Published:** 2014

**Authors:** Saima Naseem, Khursheed Hashmi, Fatima Fasih, Shaheen Sharafat, Rafiq Khanani

**Affiliations:** 1 Saima Naseem, MBBS, M.Phil, Pathology Department, Dow University of Health Sciences, Dow University of Health Sciences, Karachi, Pakistan.; 2 Khursheed Hashmi, MSc, PhD, Pathology Department, Liaquat National Hospital& Medical College, Karachi, Pakistan.; 3 Fatima Fasih, MBBS, M.Phil, Shaheen Sharafat, MBBS, M.Phil, PhD, Pathology Department, Dow University of Health Sciences, Dow University of Health Sciences, Karachi, Pakistan.; 4 Shaheen Sharafat; 5 Rafiq Khanani, MBBS, M.Phil, PhD, Pathology Department, Dow University of Health Sciences, Dow University of Health Sciences, Karachi, Pakistan.

**Keywords:** *Azadirechta indica*, Dental caries, Miswak, *Salvadora persica*

## Abstract

***Background and Objective: ***Miswak is a natural tooth cleaning tool which is being used in many parts of the world since ancient times. It is known to be useful in prevention of dental caries. But still it is not used as frequently as other oral hygiene tools. This research was designed to scientifically establish antimicrobial effect of miswak in vitro against common oral pathogens.

***Methods: ***This was a cross-sectional study involving 100 health care workers. This research was carried out in Microbiology section of Dow Diagnostic Research & Reference Laboratory. A questionnaire was designed to test oral hygiene habits of study subjects. Oral swabs were taken and microorganisms were identified by standard bacteriological methods. Test material included four different types of miswaks i.e. (1) root of the peelu (*Salvadora persica)* tree (in packing) (2) root of the peelu tree (without packing) (3) stem of the peelu tree & (4) stem of the neem (*Azadirechta indica) *tree. These miswaks were tested against three different types of microorganisms isolated from oral swabs: *Staphylococcus aureus*, *Streptococcus mutans *& *Candida albicans* by agar diffusion method. Inhibition zone was measured after 24 hrs of incubation at 37^o^C.

***Results: ***Among the miswaks used, root of the peelu tree in both packing and without packing exhibited strong antimicrobial effect against all three tested microorganisms. However miswak taken from the stem of the peelu and neem tree did not show any antimicrobial activity against all three types of the tested microorganisms.

***Conclusion: ***Miswak taken from the root of the peelu tree exhibited antimicrobial activity against all the common oral pathogens and could be a good oral hygiene tool in combating dental caries.

## INTRODUCTION

Dental caries is one of the main oral problems suffered by the human population. It is a very destructive disease of teeth.^1^ It causes decomposition of teeth by the eroding action of the acids released by the fermentation of food particles left on teeth.^2^ Oral microorganisms such as *Streptococcus mutans, Lactobacilli acidophilus, Staphylococcus aureus *&* Candida albicans etc. *build up a thick whitish layer on the tooth by combining with the remaining food debris and saliva known as dental plaque.^3^ It releases acid which causes destruction of the tooth surface and form holes and cavities in the teeth.^4^

Poor oral hygiene due to lack of proper knowledge of dental care has led to increased prevalence of dental caries around the world. Its treatment is costly and requires the expertise of highly skilled professionals. In developing countries where dental care facilities are out of reach of most of people, there is need to educate them about dental care and to promote traditional means of teeth cleaning. As far as natural oral hygienic tools are concerned, miswak is considered to be the first tool which was used by mankind as early as 5000 BC.^5^ It is still used in many parts of the world especially Muslim countries.^6^ It is taken either from twigs, stems or roots of variety of plant species.^7^ Miswak which are commonly used in Pakistan are Peelu (*Salvadora persica*), Neem (*Azadirechta indica*), Zaitoon (*Olea europaea*), Kikar (*Accacia arabica*), Ban (*Glycosmic pentaphylla*), Khiran (*Capparis aphylla*).^6^ Among these the most commonly used miswak is peelu.^7^ Research studies shows that miswak contains certain natural chemical compounds which plays an important role in maintaining good oral hygiene.^8^ These compounds are alkaloids (salvadorine), benzylisothiocyanate (BIT), calcium, chloride, essential oils, fluoride, resin, silica, sulfated compounds, tannins, salicylic acid, sterol, trimethylamine, saponins & flavenoids.^7^^,^^8^ It costs little and is easily available in many parts of the world.^7^ In the year 2000 an international consensus report on oral hygiene concluded that miswak can be a good oral hygiene tool and further research should be done to test its efficacy against oral pathogens.^8^

Today use of miswak is not as common as it should be after such an influential emphasis. There is need to convince people to use it as an oral hygiene tool. This study would be a step forward to highlight natural antimicrobial effects of miswak.

## METHODS

***Subject selection: ***Hundred subjects were included in the study. These subjects were health care workers (doctors & laboratory technicians) of DUHS who had not taken any antibiotic in the last two weeks. They were not suffering from any obvious health problem & were apparently healthy. They were not having smoking or other tobacco product using habits. 

***Collection of sample: ***Oral hygiene habits of the subjects were probed by means of a questionnaire. Subjects were trained to swab their teeth before cleaning in the morning & bring the swab with them when they reported for duty. These swabs were then inoculated on blood agar, chocolate agar and sabouraud’s agar. Microorganisms were identified according to the standard bacteriological methods.

***Selection of miswak: ***Four different types of miswaks were purchased from the local market i.e. root of the peelu tree in packing (M1), root of the peelu tree without packing (M2), stem of the peelu tree (M3)& stem of the neem tree (M4). Miswak from the root of the peelu tree is available in the market in two forms i.e. in packing with a brand name & without packing. Packed form is the one which is vacuum packed by some reputed companies after cleaning it in order to give hygienic form of the miswak. Both types were used in the study to see any difference in their antimicrobial activity. All of these miswaks were standardized by cutting with the help of a cutter into two circular pieces weighing 0.2g & 0.3g.

***Antimicrobial testing of miswak: ***Antimicrobial testing of miswak was done by agar diffusion method. All four types of miswak pieces having equal weight of 0.2g and 0.3g were placed on two separate MHA plates (for *Staphylococcus aureus* and *Candida albicans*) & on Blood agar plate (for *Streptococcus mutans*) inoculated with respective microbial lawn culture along with positive control (antibiotic / antifungal drug) & negative control (normal saline). Vancomycin disc 30µg was used as a positive control against isolates of *Streptococcus mutans* and *Staphylcoccus aureus*. Amphotericin B 10 µg /ml was used as a positive control against isolates of *Candida albicans*. 20µl of amphotericin B was poured into a well of 4mm diameter made by sterilized cork borer. Normal saline was used as a negative control by pouring 50 µl into a well of 4mm. These MHA plates were then incubated at 37^o^ C for 24hrs for the growth of *Staphylococcus aureus* and *Candida albicans* and blood agar plates were placed in CO_2 _incubator at 37^o^ C for the growth of *Streptococcus mutans*. After 24 hrs, inhibition zones were measured in mm. All tests were performed in triplicate and mean values were taken. Statistical analysis was done by SPSS.

## RESULTS

Out of 100 study subjects, 43% were males and 57% were females. Mean age of the participants was 32.5 years ± 5.679 SD (range: 24 - 45).

Among these 100 subjects, 90 were tooth-brush users, 10 were both toothbrush & miswak users and none of them were only miswak users. Among the 10 subjects who were both miswak & toothbrush users, seven were using peelu miswak, two were using neem miswak and one was using kikar miswak. It was also found that among these, three were using packed form of miswak and seven were using unpacked form of miswak. Moreover amongst the same 10 subjects, who were using both tooth brush & miswak, two were regular miswak users, three were infrequent users and five were the ones who were using miswak only during fasting.

According to the experimental part of the study, from 100 oral samples isolated, 97 were *Staphylococcus aureus*, 40 were *Streptococcus mutans*, 32 were *Candida albicans*, two were *Pseudomonas aeruginosa* and one was *Klebsiella* species. Moreover, out of these 100 samples, 43 were monomicrobes and 57 were polymicrobes. Among these isolated microorganisms only *Streptococcus mutans*, *Staphylococcus aureus *& *Candida albicans* were tested against miswak as the study was to test antimicrobial activity of miswak against common oral pathogens.

In-vitro antimicrobial activity of miswak showed that both M1 and M2 miswak strongly inhibited all three types of the isolated microorganisms however M3 and M4 miswak did not show any activity against the tested microorganisms.

***Statistical analysis: ***Statistical analysis of the data was done by SPSS version 17. One way ANOVA and Post hoc tukey test was used for the interpretation of results. Among the tested microorganisms *Candida albicans* was found to be the most sensitive to both M1 and M2 miswak while *Staphylococcus aureus* was less affected and *Streptococcus mutans* the least. Percentage of the sensitive microorganisms against four different types of the miswaks is shown in the Table-I.

**Table-I:** Microorganisms sensitive to antimicrobial components of miswak (Percentage)

*Microorganisms*	*Type of miswak*			
	*M1* *Root of the Peelu tree* a	*M2* *Root of the Peelu tree* b	*M3* *Stem of the Peelu tree*	*M4* *Stem of the Neem tree*
Streptococcus mutans N = 40	85%	82%	0%	0%
Staphylcoccus aureus N = 97	86%	89%	0%	0%
Candida albicans N = 32	87%	90%	0%	0%

a In packing,

b Without packing

When mean inhibition zones of miswak were compared against three types of the isolated microorganisms, it was found that both M1 and M2 miswak exhibited highest mean zone of inhibition against *Candida albicans* in comparison to *Staphylococcus aureus* and *Streptococcus mutans*. Moreover when mean inhibition zones of both M1 & M2 miswak at 0.2gm were compared with mean inhibition zone of their respective positive control against the three selected microorganisms, it was found that mean inhibition zone of both M1 and M2 miswak at 0.2gm were comparable to each other as well as to their respective positive controls.

However, when mean inhibition zone of both M1 and M2 miswak at increasing weight of 0.3gm was compared with positive control, it was found that both M1 and M2 miswak exhibited larger zone of inhibition in contrast to their respective positive control. This revealed that increasing the weight of the miswak by cutting thicker piece enhanced its activity against the microorganisms. These findings are illustrated in the Table-II.

**Table-II:** Mean inhibition zone of miswak against microorganisms

*Microorganism*	*Statistical variables*	*Types of miswak*								
		*M1* b		*M2* c		*M3* d		*M4* e		*Positive control*
		*0.2g*	*0.3g*	*0.2g*	*0.3g*	*0.2g*	*0.3g*	*0.2g*	*0.3g*	
Streptococcus mutans	Mean inhibition zone (mm± SD^a^)	20.4± 6.40	26.5± 8.63	19.8± 7.34	25.8± 9.73	0.0± 0.0	0.0± 0.0	0.0± 0.0	0.0± 0.0	21.7± 2.92
	Range (mm)	0-32	0-30	0-38	0-37	0.0	0.0	0.0	0.0	17-27
Staphylococcus aureus	Mean inhibition zone (mm± SD^a^)	24.9± 8.18	30.7± 9.74	25.7± 7.84	31.2±8.85	0.0± 0.0	0.0± 0.0	0.0± 0.0	0.0± 0.0	25.9± 5.49
	Range (mm)	0-37	0-36	0-41	0-42	0.0	0.0	0.0	0.0	15-36
Candida albicans	Mean inhibition zone (mm± SD^a^)	26.4± 10.68	33.0±12.88	26.2±9.35	32.8± 11.28	0.0± 0.0	0.0± 0.0	0.0± 0.0	0.0± 0.0	26.5± 5.17
	Range (mm)	0-36	0-35	0-41	0-44	0.0	0.0	0.0	0.0	20-38

a S.D.: Standard deviation,

b M1: Root of peelu tree (in packing),

c M2: Root of peelu tree (without packing)

d M3: Stem of peelu tree,

e M4: Stem of neem tree

Statistically when mean inhibition zone of both M1 & M2 miswak at 0.2g were compared with mean inhibition zone of their respective positive control against the three target microorganisms, it was found that they were identical to their respective positive controls with statistically insignificant p-value. It shows that at 0.2g both types of miswak i.e. M1 and M2 exhibited equivalent zone of inhibition with the positive control. However when mean inhibition zone of miswak at increasing weight of 0.3gm was compared with positive control, both M1 and M2 miswak exhibited larger zone of inhibition in contrast to their respective positive control with statistically significant p-value. This shows that increasing the weight of the miswak by cutting thicker piece enhances its activity against microorganisms. At the same time when inhibition zones of 0.2g and 0.3g of M1 miswak were compared with 0.2g and 0.3g of M2 miswak, no significant p-value was found. This shows that both M1 & M2 miswak exhibited almost similar zone of inhibition at equal weights. As M3 miswak and M4 miswak did not exhibit any antimicrobial activity, so statistically significant difference was found when M1 miswak & M2 miswak were compared with M3 & M4 miswak at both 0.2 and 0.3g. All of these findings are illustrated in Table-III.

**Table-III:** Statistical analysis of zone of inhibition of different types of miswak at 0.2 g & 0.3g against microorganisms

*Microorganisms*	*P- value*							
	*M1 vs M2*		*M1 vs PC*		*M2 vs PC*		*M1 & M2 vs* *M3 & M4*	
	*0.2 g*	*0.3 g*	*0.2 g*	*0.3 g*	*0.2 g*	*0.3 g*	*0.2 g*	*0.3 g*
Staphylococcus aureus	.868	.986	.740	.000*	.999	.000*	.000*	.000*
Streptococcus mutans	.973	.980	.705	.004*	.325	.024*	.000*	.000*
Candida albicans	1.000	1.000	1.000	.013*	1.000	.018*	.000*	.000*

M1: Root of peelu tree (in packing), M2: Root of peelu tree (without packing)

M3: Stem of peelu tree, M4: Stem of neem tree, PC: positive control,

PC for Staphylococcus aureus & Streptococcus mutans: Vancomycin

PC for Candida albicans: Amphotericin B,

## DISCUSSION

The highest percentage of the microorganism isolated from oral swabs taken from study subjects was of *Staphylococcus aureus*, then of *Streptococcus mutans* and *Candida albicans *respectively. These results were consistent with the study done by Al Bayati et al^8^ & Sher et al.^9^

All of the miswaks which were collected for the study were kept at room temperature instead of keeping at -80^o^ C as done by Sofrata et al^10^ in order to see the effects of miswak in its natural environment. Moreover the outer layer of the miswak was not removed during testing its antimicrobial activity which was in contrast to Safrata et al^10^ study in which they removed outer layer of the miswak before cutting with the thought that may be outer layer of the miswak is contaminated with certain bacteria and can affect the results of the study. Antimicrobial activity of miswak was observed in the present study even when its outer layer was not removed. This shows that chemical ingredients of miswak are strong enough to kill any bacteria present on its outer layer.

The hypothesis of the present study that miswak has antimicrobial activity against oral pathogens was proved by the results of the study. However sensitivity of four types of the miswak against oral microorganisms was not similar. Miswak from the root of the peelu (*Salvadora persica*) tree in both packed and unpacked form was found to be strongly active to all three types of oral microorganisms, however the percentage of the sensitivity of each varied. These results are consistent with the previous studies^10^^,^^11^^-^^13^ but in contradiction with the study of Almas^6^ in which aqueous extract of peelu miswak did not show any activity on *Streptococcus mutans*, *Staphylococcus aureus* and *Candida albicans*. This difference may be due to the various strains and type of method used.^8^

The mean inhibition zones produced by the miswak pieces used in this study against all three types of microorganisms were quite large. For example, mean inhibition zone of 0.2 and 0.3g of M1 miswak against *Candida albicans* was 26.4 mm and 33.0 mm respectively which was consistent with the results of the study done by Sofrata et al.^10^ These findings support the hypothesis of the Sofrata et al^10^study that miswak in the stick form has more powerful inhibitory effect against most of the microorganisms than the miswak extract which usually exhibited small inhibition zone. For example aqueous extract of peelu miswak produced zone of inhibition of 12.4 mm at 200%, 11.3mm at 100% and 10.5mm at 50% concentration against *Candida albicans* in a study done by Al Bayati et al.^8^ In another study by Almas et al^14 ^a growth inhibition zone of about 3mm was achieved by 50 % concentration of aqueous extract of peelu miswak on *Streptococcus mutans*.

Antimicrobial activity of the miswak was increased with increase in weight. For example M1 miswak exhibited mean inhibition zone of 24.9mm at 0.2g and 30.7mm at 0.3g against *Staphylococcus aureus* as shown in Table-II. These results were consistent with the Sofrata et al^10^ study in which activity of the miswak increased with increase in weight.

The sensitivity (mean inhibition zone) of both M1 and M2 miswak against all three types of the isolated microorganisms was also not similar, which may be due to the difference in the release of the constituents of miswak, variance in the membrane permeability of the microorganism, inactivation of the contents of miswak or change in its pH as concluded by Al Bayati et al^8^, Sher et al^9^ and Almas et al.^6^

The pH plays a vital role in the activity of miswak as seen by Almas et al^6^ in his study of evaluating antimicrobial activity of seven different chewing sticks. According to his findings functional pH ranged from 5.7- 6.1 and value above this did not show any activity against microorganisms. Rani et al^15^ also found in their study that antimicrobial activity of miswak differ among themselves and is microorganism specific.

While comparing M1 miswak with M2 miswak, statistical analysis shows that zone of inhibition of these two miswaks were comparable at two standard weights. This shows that packing does not lead to a major difference in the activity of miswak. It is the chemical constituents of miswak which exhibit antimicrobial activity against microorganisms.

The other two types of miswak i.e. M3 miswak and M4 miswak did not show any activity against all the three types of the isolated microorganisms. However other studies have reported antimicrobial activity in extracts of miswak from stem of neem and peelu tree.^1^^,^^8^^,^^16^

During the collection of the oral samples, subjects were also asked about their oral hygiene habits. Out of 100 of our subjects 90 were tooth brush users, 10 were both tooth brush and miswak users and none of them used miswak alone. These results were consistent with the National health survey of Pakistan (1990-1994), which showed that greater portion of the urban population use tooth brush for cleaning their teeth.^17^

Continuous new developments in science and technology have led most of the people to perceive use of miswak as an old custom. However its use should be encouraged with scientific reasoning. Further research studies should be carried out to see the basic mechanism of the activity of miswak against microorganisms. It is important to see whether antimicrobial effect of miswak is due to the cumulative effect of all of its chemical constituents or individually they can exhibit the similar effects. This can be very useful in manufacturing of antimicrobial drugs. 

Moreover there is need to do clinical trials on large scale on the local population to see the effects of miswak in comparison with toothpaste and tooth brush as so far locally no such data is present. It is also necessary to educate the people of Pakistan about the medicinal properties of miswak as its urban population is still hesitant to use it on regular basis.

## CONCLUSIONS

Antimicrobial activity of miswak is as good as any antibiotic or antifungal drug and with increasing weight of the miswak its activity also increases.

## Notes

***Funded: ***This research project was funded by Dow University of Health Sciences.
